# A concept of equivalent‐volume concordance coefficient for comparing target volumes in radiotherapy

**DOI:** 10.1002/acm2.70611

**Published:** 2026-05-14

**Authors:** Xue Ou, Xianfei Qin, Huaye Wei, Lianrong Zheng, Di Huang, Qiulu Zhong, Kai Hu, Qinghua Du

**Affiliations:** ^1^ Department of Radiation Oncology The First Affiliated Hospital of Guangxi Medical University Nanning China; ^2^ Department of Radiation Oncology The Second Affiliated Hospital of Guangxi Medical University Nanning China

**Keywords:** concordance coefficient, equivalent‐volume, radiotherapy, target volume

## Abstract

**Background:**

The significant limitation of simple volume analyses for target volumes is that they disregard the spatial distribution of non‐overlapping volumes and the surrounding dose gradient.

**Purpose:**

To present a concept of equivalent‐volume concordance coefficient (*CC*_EV_) for comparing target volumes in radiotherapy, which takes into account the dose coverage requirement and the spatial relationship between the standard volume (*V*_Std_) and the reference volume (*V*_Ref_).

**Methods:**

By using uniform expansion and Boolean operations, the non‐overlapped volumes were segmented by a certain spatial distance (*d*) from the margin of *V*_Std_ or *V*_Ref_. The volume of *V*_Std‐I_ and *V*_Ref‐I_ were defined as *V*_Std_ and *V*_Ref_ minus their intersection volume (*V*_I_), respectively. The equivalent volume (EV) increased with *d* (*EV*_Std‐I _= ∑*V*_Std‐I__ *_i_* (*d*_i_/*d_r_*)*^n^*; *EV*_Ref‐I _= ∑*V*_Ref‐I__ *_i_* (d*_i_*/*d_r_*)*^n^*). The dose‐volume histograms (DVHs) of whole body were exported in 20 volumetric modulated arc therapy (VMAT) plans. The radius (R) of the equivalent sphere for *V*_PCT_ (the volume covered by a percentage dose (PCT)) was calculated (*R*_PCT _= (3*V*_PCT_/4π)^1/3^), and the mean dose fall‐off distance (*d*_PCT_) from *V*_PCT_ to *V*_100%_ was calculated by the equation: *d*_PCT _= *R*_PCT_‐*R*_100%_. The distance‐penalty parameter n was determined by the exponential relationship between PCT and *d*_PCT_. The *CC*_EV_ was evaluated via the Jaccard coefficient (JC) and dice similarity coefficient (DSC) and was compared against traditional indices in an illustrative case and two sets of volumes delineated at different time points.

**Results:**

The DVH could be divided into prescription dose region, rapid fall‐off region, slow fall‐off region and low‐dose region according to dose gradients of 20 plans. The volume of *V*_48%_ minus *V*_100%_ was contained in the rapid dose fall‐off region, where the mean *d*_48%_ was 2.03 cm (1.75–2.40 cm), and PCT was linearly correlated with *d*_PCT_ (all *R* ^2^≥0.987, *P* < 0.001), leading to the setting of reference distance (*d*_r_) = 1 cm and *n* = 1. *CC*_EV_ distinguished different intersection conditions and yielded reasonable results. The mean *JC*_EV_ and *DSC*_EV_ of two volume sets were 0.929 and 0.963, respectively.

**Conclusions:**

A reference penalty distance of 1 cm and a distance‐penalty parameter of 1 are recommended for *CC*_EV_ in clinical practice. *CC*_EV_ effectively incorporates the spatial location of non‐overlapped volumes and key radiotherapy requirements.

## 1 Introduction

With the development of technologies in tumor diagnosis, staging and radiation delivery, the target volumes in radiotherapy have evolved from two‐dimensional target volume to highly conformal three and four‐dimensional target volumes.^1^ One of the major purposes of conformal radiotherapy is to adequately cover the target volume with sparing of surrounding tissue and organs at risk.^2^ It is absolutely necessary to determine the exact location and extent of the tumor. Novel imaging techniques^3^, ^4^ and automatic segmentation technology^5^, ^6^ may improve the delineation of target volume, but its accuracy should be taken into account before clinical application. Analysis of delineation variation and accuracy is necessary to assess the potential improvements that new techniques or technologies may bring to target volume definition.^7^

Geometrical volume analysis is widely used for comparison of target volume delineations. A good deal of methods of volume analysis have been proposed, such as simple volume analysis,^8^ center of mass analysis,^9^ concordance index type analysis,^10^, ^11^, ^12^ and volume edge analysis.^13^ Simple volume assessment is a widely reported method of comparing radiotherapy volumes, but the obvious disadvantage is that different intersection cases are not considered. Another popular method is the concordance index, also referred to as the conformity index (CI). The classic versions of this method are the Jaccard coefficient (JC) and the dice's similarity coefficient (DSC).^12^ As a single measurement, the concordance index provides more information on both intersection volume and non‐intersection volume, but it cannot distinguish the spatial position of two volumes or the reasons for variation. In addition, most of the geometric analyses deal only with the concept of geometry, focusing on similarity or overlap rate without specifically considering the requirements of radiotherapy. The purpose of the present work is to define a simple equivalent‐volume concordance coefficient (*CC*_EV_) which takes into account the dose coverage requirement and the spatial relationship between the two volumes.

## 2 Method

### 2.1 Definition of *CC*_EV_

The concept of equivalent volume (EV) is introduced to account for the clinical significance of non‐overlapped volumes based on their distance from the margins of the standard volume (*V*_Std_) and the reference volume (*V*_Ref_). The intersection volume of *V*_Std_ and *V*_Ref_ is defined as V_I_. The volumes *V*_Std_ minus V_I_ and *V*_Ref_ minus V_I_ are defined as *V*_Std‐I_ and *V*_Ref‐I_ respectively. Assuming *V*_Ref_ represents the treatment volume, the required dose coverage differs for non‐overlapped volumes depending on their proximity to *V*_Std_. This differential requirement is quantified by the EV. A smaller EV is assigned to *V*_Std‐I_ near *V*_Ref,_ as this under‐covered volume would still receive a relatively acceptable, albeit suboptimal, high dose. Similarly, a smaller EV is assigned to *V*_Ref‐I_ near *V*_Std_, as this over‐covered volume receiving the prescribed dose is relatively acceptable, given that it would have received a suboptimal high dose if *V*_Std_ were the treatment volume. The equivalent volume of *V*_Std‐I_ (*EV*_Std‐I_) is supplied by the equation:

(1) $$EV_{\mathrm{Std}-I}=\sum V_{\mathrm{Std}-I\_i}{\left(d_{i}/d_{r}\right)}^{n},$$

and the equivalent volumes of *V*_Ref‐I_ (*EV*_Ref‐I_) is supplied by the equation:

(2) $$EV_{\mathrm{Ref}-I}=\sum V_{\mathrm{Ref}-I\_i}{\left(d_{i}/d_{r}\right)}^{n}.$$

**FIGURE 1:**
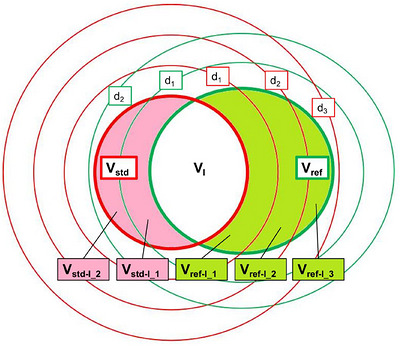
Illustration of volumes segmented outside the intersection volume in equivalent‐volume model (standard volume, *V*_Std_; reference volume, *V*_Ref_; intersection volume of *V*_Std_ and *V*_Ref_, *V*_I_; the volume of *V*_Std_/*V*_Ref_ minus *V*_I_, *V*_Std‐I_/*V*_Ref‐I_; the ith sub‐volume of *V*_Std‐I_/*V*_Ref‐I_,*V*_Std‐I_i_/*V*_Ref‐I_i_; the distance from *V*_Std‐I_i_/*V*_Ref‐I_i_ to *V*_Ref_/*V*_Std_, *d_i_*).

Here, *V*_Std‐I__ *_i_* (or *V*_Ref‐I__ *_i_*) is the *i*th sub‐volume of *V*_Std‐I_ (or *V*_Ref‐I_), *d*_i_, with 0.1 cm as the unit distance, is the distance from this subvolume to *V*_Ref_ (or *V*_Std_) (Figure 1), *d*_r_ is a reference distance for which no equivalent conversion is applied, and n is the distance‐penalty parameter. The parameter n reflects the dose‐distance relationship: n=1 when the dose depends linearly on distance; *n* = 2 when it depends on the square of the distance; and *n* = 0 when the distance penalty is disregarded. In this study, *d*_r_ was set to 1 cm for the following reasons: (1) 1 cm is a basic distance unit, and the calculation of the distance ratio is convenient and easy to understand; (2) our previous study found that the dose gradient in the 2 cm range was roughly similar; (3) given that most target volume deviations do not exceed 2 cm,^14^, ^15^, ^16^, ^17^, ^18^ a primary monitoring distance of approximately 2 cm can be considered reasonable in clinical practice.

When EV model is used in JC (*JC*_EV_), the equation is as follow:

(3) $$JC_{\mathrm{EV}}=V_{I}/\left(V_{I}+\sum V_{\mathrm{Ref}-I\_i}{\left(d_{i}/d_{r}\right)}^{n}+\sum V_{\mathrm{Std}-I\_i}{\left(d_{i}/d_{r}\right)}^{n}\right).$$

Assuming the volume beyond *V*_Std_ and *V*_Ref_ is ignored, the evaluation of the new delineation method (reference volume) can be considered a diagnostic test with a true negative rate of zero (Table 1). Under this premise, the *JC*_EV_ is equal to the agreement rate (coincidence rate), and the same applies to JC.

**TABLE 1:** Diagnostic test of a new delineation method.

	Standard method		
New method	Positive	Negative	Total
Positive	*V*_I_ ^a^	*EV*_Ref‐I_ ^b^	*V*_I_ + *EV*_Ref‐I_
Negative	*EV*_Std‐I_ ^c^	0	*EV*_Std‐I_
Total	*V*_I_ + *EV*_Std‐I_	*EV*_Ref‐I_	*V*_I_+*EV*_Std‐I_ + *EV*_Ref‐I_

^a^ *V*_I_, the intersection volume of *V*_Std_ and *V*_Ref_.

^b^ *EV*_Ref‐I_, the equivalent volume of *V*_Ref_ minus *V*_I_.

^c^ *EV*_Std‐I_, the equivalent volume of *V*_Std_ minus *V*_I_.

When EV model is used in DSC (*DSC*_EV_), the equation is as follow:

(4) $$\begin{array}{cc} DSC_{\mathrm{EV}} & =2V_{I}/\left(2V_{I}+\sum V_{\mathrm{Ref}-I\_i}{\left(d_{i}/d_{r}\right)}^{n}\right.\\ & \;\left.+\sum V_{\mathrm{Std}-I\_i}{\left(d_{i}/d_{r}\right)}^{n}\right). \end{array}$$

When *n* = 0, *JC*_EV_ is equal to JC, and *DSC*_EV_ is equal to DSC. When 95% of the volumes of *EV*_Ref_ and *EV*_Std_ overlap, the target volumes are considered essentially consistent (i.e., the discrepancy is a low‐probability event). Under the condition of equal target volume sizes, the *JC*_EV_ and *DSC*_EV_ are 0.90 and 0.95, respectively (rounded to two significant figures). Values below these thresholds indicate a significant difference between the target volumes.

### 2.2 Estimation of distance‐penalty parameter

Gross tumor volumes (GTVs) were delineated for 20 nasopharyngeal carcinoma cases, and the treatment plans were designed with volumetric modulated arc therapy (VMAT). All plans were designed and optimized using the inverse treatment planning system (software version: Varian Eclipse 13.6). The planning target volume (PTV) for the GTV was prescribed a dose of 70 Gy. The dose‐volume histogram (DVH) of the whole body was exported from each plan, from which the dosimetric parameters of *V*_PCT_ (the volume covered by a percentage dose (PCT)) were extracted. The radius (R) of the equivalent sphere for each *V*_PCT_ was calculated by the equation^19^:

(5) $$R_{\mathrm{PCT}}={\left(3V_{\mathrm{PCT}}/4\pi \right)}^{1/3},$$

and the mean distance from each *V*_PCT_ isodose line to the *V*_100%_ isodose line could be calculated by the equation:

(6) $$d_{\mathrm{PCT}}=R_{\mathrm{PCT}}-{R_{100}}_{\%}.$$

Finally, the distance‐penalty parameter n was determined by the exponential relationship between PCT and *d*_PCT_.

### 2.3 Comparison of *CC*_EV_ with other conformity indices in an illustrative case

**FIGURE 2:**
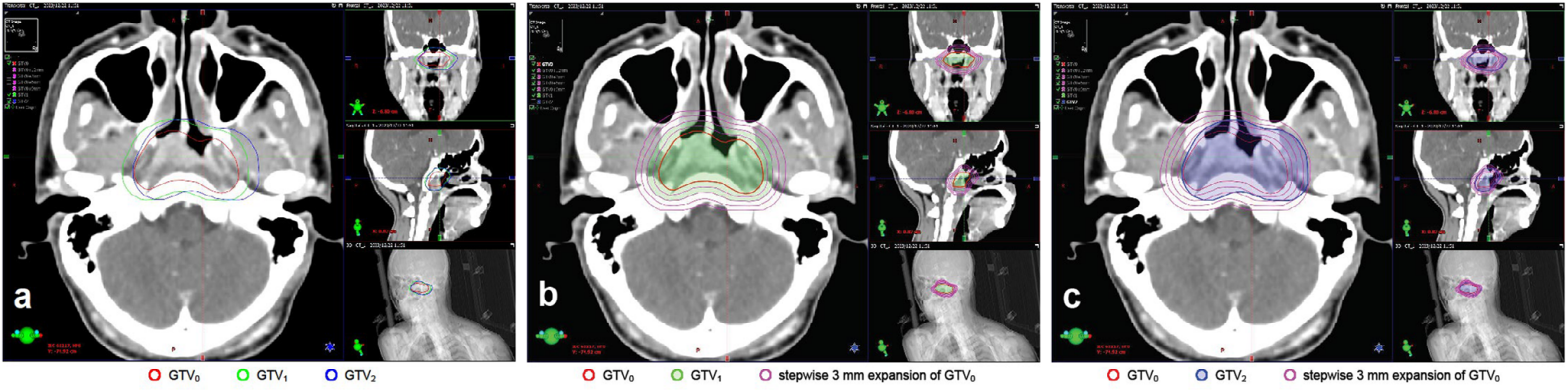
(a) *GTV*_0_ was the standard volume, *GTV*_1_ was a reference volume generated by uniformly expanding *GTV*_0_, and *GTV*_2_ was a reference volume of the same size as *GTV*_1_ but with a nonuniform spatial relationship to *GTV*_0_; (b) The volume of *GTV*_1_ outside *GTV*_0_ was divided in 3 mm increments; (c) The volume of *GTV*_2_ outside *GTV*_0_ was divided in 3 mm increments.

Three GTVs were contoured for a representative case to facilitate the comparison: (a) *GTV*_0_, defined as the *V*_Std_; (b) GTV_1_, a reference volume generated by uniformly expanding *GTV*_0_; and (c) GTV_2_, a reference volume of the same size as *GTV*_1_ but with a nonuniform spatial relationship to GTV_0_. Using uniform expansions (in 0.3 cm increments) and Boolean operations, both *V*_R‐I__ *_i_* and *V*_S‐I__ *_i_* were calculated in *GTV*_0_and*GTV*_1_ group and *GTV*_0_and*GTV*_2_ group respectively (Figure 2a–2c). *JC*_EV_, *DSC*_EV_, JC and DSC in two groups were then calculated. In the case of non‐unit distances, *d_i_* is calculated by the average distance. For example, if the volume within a 0.3 cm range contains distances of 0.1 cm, 0.2 cm, and 0.3 cm, the average d*_i_* is calculated as (0.1 cm + 0.2 cm + 0.3 cm)/3.

### 2.4 Clinical application of *CC*_EV_ in target volume comparison

The GTVs of 20 NPCs were delineated twice by a same senior radiation oncologist at two different time points (with an interval of no less than 3 months), resulting in two sets of GTVs (*GTV*_pre_ and *GTV*_post_). The non‐overlapping volumes were segmented with a spatial resolution of 0.3 cm, and *JC*_EV_, *DSC*_EV_, JC and DSC were calculated for the two sets of GTVs. The differences in coefficients between the EV mode and the conventional mode were evaluated, along with the coefficients under the premise that the target volume delineation methods were essentially consistent. To preliminarily assess the impact of spatial resolution, the *JC*_EV_ and *DSC*_EV_ were recalculated using a spatial resolution of 0.6 cm.

### 2.5 Statistical analysis

SPSS 19.0 was used for statistical analysis. The relationship between PCT and *d*_PCT_ was assessed by linear regression, followed by curve estimation when a linear fit was inadequate. The relationship between PTV size and the resulting regression coefficient was assessed by Pearson correlation analysis. The variation in regression coefficient were analyzed by coefficients of variation (CV). Comparisons between different indices were performed using the paired t‐test.

## 3 RESULT

### 3.1 Analyses of dose gradient and distance‐penalty parameter

**FIGURE 3:**
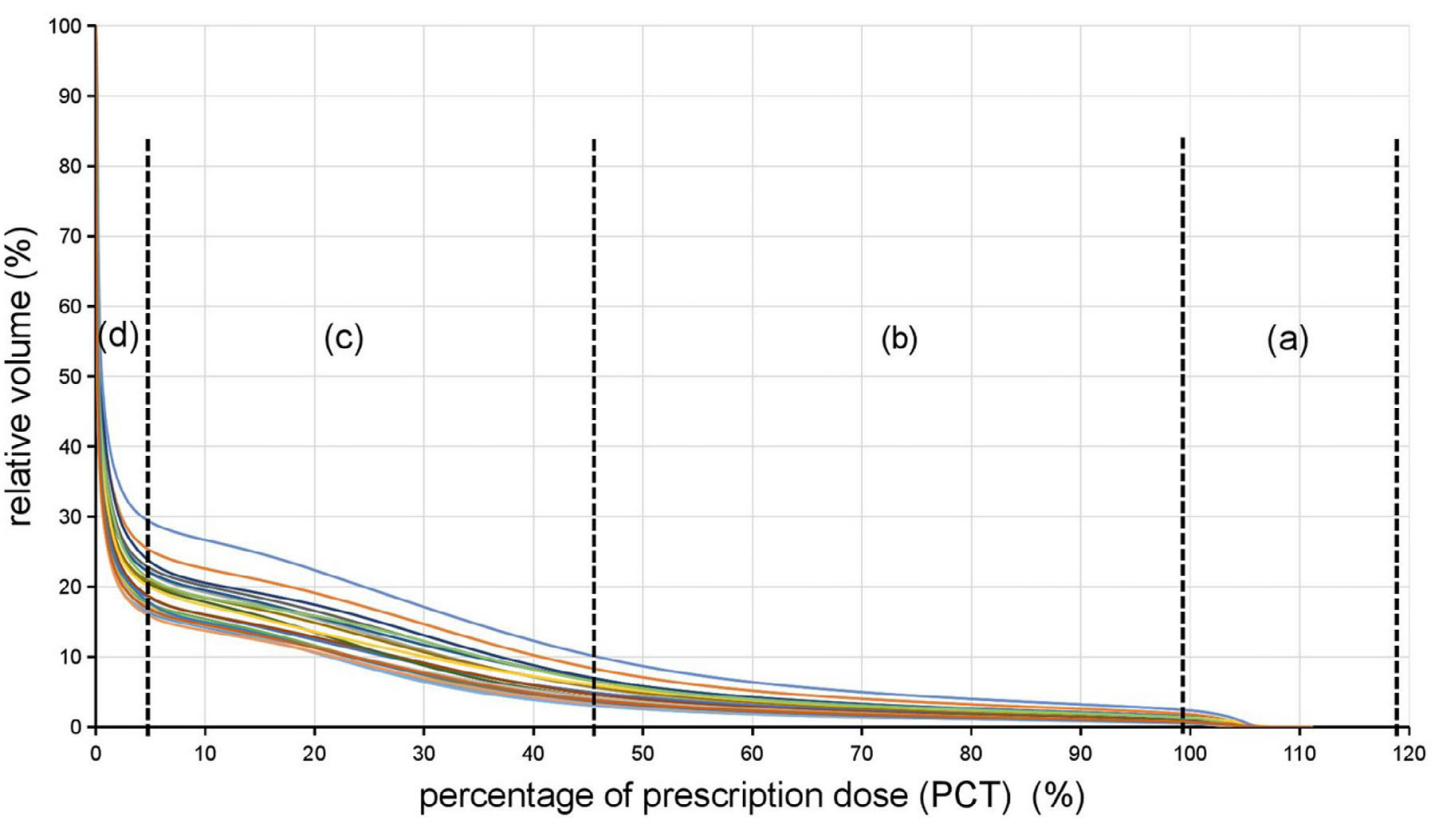
DVHs of VMAT could be divided into four regions (from 20 cases): (a) prescription dose region; (b) rapid fall‐off region; (c) slow fall‐off region; and (d) low‐dose region.

**FIGURE 4:**
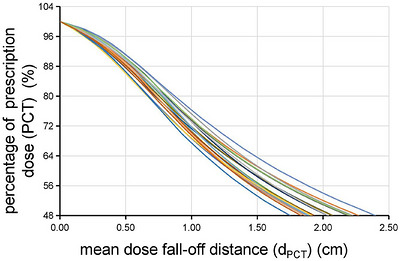
Percentage of prescription dose (PCT) as the mean dose fall‐off distance (*d*_PCT_) changed (from 20 cases).

Based on the dose gradient observed across the 20 cases, the DVHs could be categorized into four distinct regions: (a) prescription dose region; (b) rapid fall‐off region; (c) slow fall‐off region; and (d) low‐dose region (Figure 3). While the percentage dose (PCT) exhibited an approximately linear relationship with volume in both the rapid and slow fall‐off regions, their slopes differed significantly. The volume between the *V*_48%_ and *V*_100%_ isodose lines laid within the rapid fall‐off region, and the average *d*_48%_ was 2.03 cm (1.75–2.40 cm) (Figure 4).

The linear relationship between PCT and *d*_PCT_ was validated by the model (PCT = *B*_0_+*B*_1_ *d*_PCT_), which yielded *R* ^2^ values ≥ 0.987 (*P* < 0.001) for all cases (Table 2). Based on this demonstrated linearity and the clinical observation that most target volume deviations occur within the spatial extent of this rapid dose falloff,^14^, ^15^, ^16^, ^17^, ^18^ the distance‐penalty parameter *n* was assigned a value of 1.

**TABLE 2:** Linear regression results and PTV volumes of 20 cases.

Case	B0 ± SE	B1 ± SE	*R* ^2^	*P*	Volume of PTV (cm^3^)
1	102.835 ± 0.604	−30.853 ± 0.570	0.992	<0.001	58.5
2	100.209 ± 0.677	−24.880 ± 0.553	0.989	<0.001	188.7
3	102.212 ± 0.664	−26.104 ± 0.538	0.993	<0.001	99.8
4	101.796 ± 0.529	−29.971 ± 0.498	0.993	<0.001	54.4
5	100.640 ± 0.566	−30.156 ± 0.554	0.992	<0.001	97.8
6	102.823 ± 0.629	−30.054 ± 0.578	0.991	<0.001	67.0
7	100.037 ± 0.608	−26.939 ± 0.540	0.990	<0.001	143.6
8	101.647 ± 0.616	−29.672 ± 0.576	0.991	<0.001	83.6
9	100.954 ± 0.650	−26.042 ± 0.544	0.989	<0.001	135.9
10	100.935 ± 0.637	−27.477 ± 0.563	0.990	<0.001	123.8
11	101.848 ± 0.665	−24.424 ± 0509	0.989	<0.001	119.6
12	102.802 ± 0.669	−28.647 ± 0.586	0.990	<0.001	80.1
13	103.242 ± 0.605	−30.604 ± 0.559	0.992	<0.001	47.4
14	102.487 ± 0.615	−31.006 ± 0.588	0.991	<0.001	69.8
15	101.958 ± 0.521	−27.877 ± 0.454	0.993	<0.001	62.3
16	99.856 ± 0.612	−29.021 ± 0.589	0.989	<0.001	137.2
17	101.298 ± 0.670	−23.860 ± 0.510	0.989	<0.001	231.7
18	101.6511 ± 0.718	−26.193 ± 0.596	0.987	<0.001	145.5
19	101.608 ± 0.541	−32.499 ± 0.555	0.993	<0.001	46.4
20	101.909 ± 0.533	−31.098 ± 0.519	0.993	<0.001	58.7

*Note*: Data are presented as mean ± SE.

Abbreviation: PTV, planning target volume.

Further analysis revealed a strong positive correlation between the regression coefficient B_1_ (the slope of the PCT‐*d*_PCT_ relationship) and the PTV volume (Pearson *r* = 0.834, *P* < 0.001). The variability of *B*_1_ across the cohort was low, as indicated by a CV of 8.85%.

The mean regression coefficients were *B*_0_ = 101.637 and *B*_1_ = −28.369. Applying the mean model (PCT = 101.637‐28.369*d*_PCT_), PCT values corresponding to *d*_PCT_ increments of 0.2 cm from 0.2 to 1.0 cm were calculated to be 96.0%, 90.3%, 84.6%, 78.9%, and 73.3%, respectively.

### 3.2 Comparison of *JC*_EV_, *DSC*_EV_, JC and DSC

The volumes of *GTV*_0_, *GTV*_1_ and *GTV*_2_ were 19.4 cm^3^, 43.7 cm^3^ and 43.7 cm^3^, respectively. *EV*_Std_, *EV*_Ref_, *JC*_EV_, *DSC*_EV_, JC and DSC were calculated in two groups respectively. In contrast to the JC and DSC, which yielded identical values for both groups, *JC*_EV_ and *DSC*_EV_ successfully differentiated between the two spatial configurations (Table 3).

**TABLE 3:** *JC*_EV,_ *DSC*_EV_, JC, and DSC in two groups.

Group	*V*_Std_ (cm^3^)	*V*_Ref_ (cm^3^)	*V*_I_ (cm^3^)	*EV*_Std_ (cm^3^)	*EV*_Ref_ (cm^3^)	*JC*_EV_	*DSC*_EV_	JC	DSC
*GTV*_0_and*GTV*_1_	19.4	43.7	19.4	19.4	28.25	0.687	0.814	0.444	0.615
GTV_0_andGTV_2_	19.4	43.7	19.4	19.4	31.19	0.622	0.767	0.444	0.615

Abbreviations: *V*_Std_, standard volume; *V*_Ref_, reference volume; *V*_I_: the intersection volume of *V*_Std_ and *V*_Ref_; *EV*_Std_, equivalent volume of *V*_Std_; *EV*_Ref_, equivalent volume of *V*_Ref_; *JC*_EV_, equivalent‐volume Jaccard coefficient; *DSC*_EV_, EV model is used in DSC; JC, Jaccard coefficient; DSC, Dice's similarity coefficient; EV, equivalent volume; GTV, gross tumor volumes.

### 3.3 Comparison of *CC*_EV_ with other conformity indices in clinical cases

When the same radiation oncologist delineated the identical target volumes at different time points, the mean *JC*_EV_ and *DSC*_EV_ were 0.929 and 0.963, respectively, reaching the previously established concordance threshold. The corresponding JC and DSC values were 0.792 and 0.883, respectively (Table 4), which were significantly lower than their respective EV‐based indices (*P* < 0.001). Of the *GTV*_pre_ volume outside the intersection, 73.8% lay within 0.3 cm of the intersection, while for *GTV*_post_, this proportion was 74.1%. When the spatial resolution was changed from 0.3 cm to 0.6 cm, the *JC*_EV_ and *DSC*_EV_ decreased by an average of 0.019 ± 0.004 and 0.010 ± 0.002, respectively (*P* < 0.001).

**TABLE 4:** JC_EV,_ *DSC*_EV_, JC, and DSC in clinical cases of 20 NPC patients.

Case	*GTV*_pre_ (cm^3^)	*GTV*_post_ (cm^3^)	*V*_I_ (cm^3^)	JC	DSC	*JC*_EV_	*DSC*_EV_
1	38.5	42.1	35.5	0.787	0.881	0.933	0.965
2	124.3	130.7	112.3	0.787	0.881	0.928	0.963
3	65.7	71.3	60.2	0.784	0.879	0.924	0.96
4	35.8	37.4	33.0	0.821	0.902	0.945	0.972
5	64.4	58.4	53.3	0.767	0.868	0.919	0.958
6	44.1	48.9	41.8	0.816	0.899	0.944	0.971
7	94.6	86.7	79.9	0.788	0.881	0.92	0.958
8	55.1	60.6	52.1	0.819	0.901	0.943	0.97
9	89.5	85.6	78.2	0.807	0.893	0.932	0.965
10	81.5	90.0	76.3	0.801	0.890	0.929	0.963
11	78.5	82.3	71.5	0.801	0.889	0.935	0.966
12	52.7	48.2	43.0	0.743	0.852	0.913	0.955
13	31.2	33.7	29.4	0.828	0.906	0.947	0.973
14	45.6	55.0	43.2	0.753	0.859	0.918	0.957
15	41.0	39.5	36.4	0.825	0.904	0.944	0.971
16	90.3	95.9	81.6	0.780	0.876	0.924	0.96
17	152.6	132.1	117.5	0.703	0.825	0.883	0.938
18	96.0	110.8	90.2	0.774	0.872	0.913	0.955
19	30.6	32.7	28.5	0.819	0.900	0.946	0.972
20	38.7	40.5	35.9	0.829	0.907	0.949	0.974
Average				0.792	0.883	0.929	0.963

Abbreviations: *GTV*_pre_, the initial GTV delineated at the first time point; *GTV*_post_, The GTV delineated by the same radiation oncologist three months later; *V*_I_: the intersection volume of *GTV*_pre_ and *GTV*_post_; *JC*_EV_, equivalent‐volume Jaccard coefficient; *DSC*_EV_, EV model is used in DSC; JC, Jaccard coefficient; DSC, Dice's similarity coefficient.

## 4 DISCUSSION

The RTOG “conformity index” was proposed in 1993.^8^ Subsequently, several dosimetric indices for evaluating dose coverage were developed, such as the lesion coverage factor, healthy tissue CI, and conformation number.^20^ The RTOG “conformity index” is a simple index with a disadvantage of producing false perfect score in case of the same size of target volume and reference volume. However, the extreme case of inconformity of the target and prescription isodose line seems unlikely, because this index was initially proposed for radiotherapy under stereotactic conditions, a modality ensuring the most rigorous and most precise treatment planning.^21^ Furthermore, during inverse planning, strict adherence to organ‐at‐risk (OAR) constraints often takes precedence over optimizing conformity metrics. Consequently, this study focuses on applying the *CC*_EV_ to target volume comparisons, although it possesses the potential to be converted for dose coverage assessment.

JC and DSC are widely used for volume comparison, as they both account for the intersection volume. However, a significant limitation is that they disregard the spatial distribution of non‐overlapping volumes and the surrounding dose gradient. In recent years, some indices have been given more meaning than volume ratio through complex algorithms, such as normal tissue sparing index,^22^ hot and cold spots checks,^23^ normal tissue complication index,^24^ distances^25^ and prescription isodose line conformity scale.^26^ Most of the new indices developed are personalized and created using MATLAB, C‐language, and Visual basic etc. hence their application is limited to them and could not be generalized.^21^ The proposed *CC*_EV_ is fundamentally an enhanced version of the JC or DSC. Both *JC*_EV_ and JC can be conceptually derived from diagnostic test metrics and are equivalent to a coincidence rate when volumes outside the union of the structures are disregarded. In contrast, the DSC is mathematically always larger than the JC (except at the extremes of 0 and 1), indicating that JC is a more stringent metric. This makes JC, and by extension *JC*_EV_, more suitable for evaluations against a definitive standard (i.e., for accuracy). The DSC, being less penalizing, may be more appropriate for assessing similarity in contexts without a gold standard, such as comparing delineations among multiple observers.^2^

*CC*_EV_ introduced in this study is a geometrically and radiophysically motivated coefficient that integrates both spatial distance and the local dose gradient. J M Park et al. proposed a new CI based on the calculation of distances between the target volume and the volume of reference isodose,^25^ but its reliance on centroids makes it sensitive to volume shape and centroid position, often requiring supplementary metrics like standard deviation to avoid misleading scores. Meanwhile, the importance of the dose gradient has been well established in the literature,^19^, ^27^, ^28^ particularly for evaluating stereotactic radiosurgery plans.^29^ By synthesizing these two critical dimensions of spatial discrepancy and dosimetric fall‐off into a single unified concept, *CC*_EV_ provides a more comprehensive and rational framework for assessing the accuracy of novel target volume delineation methods.

VMAT, as an advanced form of IMRT, provides a highly uniform dose gradient, making it particularly suitable for analyzing the dose‐distance relationship. While the inverse square law offers a fundamental theoretical model for point sources, its direct application is complicated in clinical practice because the PTV is an extended structure, and beam penetration effects are significant. Due to the irregular shape of the target volume and the complexity of dose coverage, a uniform dose gradient is nearly impossible to achieve. When used for dose gradient studies, the equivalent sphere only reflects the overall approximate distribution^19^ and cannot represent the actual dose distribution at every distance. The dose‐distance investigation here is primarily intended to inform the selection of n between 1 and 2, rather than to derive a precise parameter through fitting. Analysis of the whole‐body DVH revealed that within the rapid dose fall‐off region (from *V*_48%_ to *V*_100%_), the PCT exhibited a strong linear correlation with the distance from the *V*_100%_ isodose surface. Although the slope of this relationship varied with PTV size, the inter‐case variability was low, and the linearity was consistently robust in all individual cases. The mean distance for *V*_48%_ was 2.03 cm, which aligns with the typical range of target volume deviations reported in clinical practice.^14^, ^15^, ^16^, ^17^, ^18^ Based on this confirmed linear relationship, a distance‐penalty parameter n of 1 is recommended for the *CC*_EV_ model, as it is physiologically justified and feasible for most clinical scenarios.

A reference penalty distance of 1 cm was adopted in this study, which indicated that the EV s at distances of 0.2, 0.4, 0.6, 0.8, and 1.0 cm were calculated as 2/10, 4/10, 6/10, 8/10, and 10/10 of their actual geometric volumes, respectively. At these distances, the observed average dose percentages were about 96.0%, 90.3%, 84.6%, 78.9% and 73.3%. The clinical reasonableness of this model is supported by the fact that a spatial error of 0.2 cm, associated with 96.0% of the target dose, is often considered more acceptable. For RTOG “quality of coverage”, if the 90% isodose covers all of the clinical and pathologic target volume, treatment is considered to comply with the protocol; and if the 80% isodose covers all of the clinical and pathologic target volume, the protocol violation is considered to be minor.^20^ It is important to note that the specific dose‐distance relationship and the measured *V*_48%_ distance presented here serve as partial, case‐specific evidence for the *CC*_EV_ concept's validity, as these parameters may vary across different clinical cases. Ultimately, the selection of a 1 cm penalty distance and a linear penalty function is grounded in common clinical practice, as it takes into account the delineation deviation extents and dose coverage requirements.

In this study, *JC*_EV_, *DSC*_EV_, JC and DSC were compared using two examples featuring reference volumes of identical size but different spatial configurations. Both *JC*_EV_ and *DSC*_EV_ in *GTV*_0_and*GTV*_1_ group were larger than those in *GTV*_0_and*GTV*_2_ group, indicating the clinical superiority of EV conformation—a distinction that JC and DSC failed to capture. For clinical applications, EV‐based indices are generally larger than conventional indices, because the volume proximal to the overlapping region is typically much larger than the volume distal to it. For instance, in this study, approximately 74% of the non‐overlapping volume was located within 0.3 cm of the overlapping region. Under conditions of consistent methodology and the same observer, the two sets of target volumes delineated at different time points can both be considered clinically acceptable for radiotherapy—that is, they are inherently highly consistent with no qualitative difference. Based on this premise, the *JC*_EV_ and *DSC*_EV_ for the target volumes from the two time points in this study were 0.929 and 0.963, respectively. Conventional JC and DSC typically lack clear thresholds^30^; this study probabilistically defines a threshold for significant differences between target volumes (0.90 for *JC*_EV_ and 0.95 for *DSC*_EV_) and demonstrates its clinical applicability—these thresholds are achieved when target volumes are essentially consistent, even though the corresponding JC and DSC values are only 0.792 and 0.883. It should be noted that these thresholds are interpreted as preliminary reference values under a specific assumption (i.e., the two target volumes are equal), rather than universal cutoffs applicable to all target‐volume comparisons.

In our preliminary study, when the spatial resolution for volume segmentation was set to 0.1 cm or 0.2 cm, the automatic contour expansion function could not reliably identify fine distances, and some voxels were lost during Boolean operations for volume segmentation. To improve the robustness and computational efficiency of the study, a spatial resolution of 0.3 cm was adopted. Further analysis showed that even when the segmentation resolution was increased to 0.6 cm, the changes in *JC*_EV_ and *DSC*_EV_ were limited, with an average reduction of less than 0.02. Therefore, until the precision of the segmentation tools is improved, using a spatial resolution of 0.3 cm represents a convenient and efficient choice.

The principal advantages of *CC*_EV_ are as follows: (a) it fully incorporates the positional information of non‐intersection volumes; (b) it integrates critical radiotherapy considerations, including local dose gradients; (c) its calculation is straightforward, relying on an intuitive formula without complex computations; (d) it serves as a versatile, general‐purpose coefficient for volume comparison, which is backward‐compatible as it can be simplified to established indices (e.g., when *n* = 0, *JC*_EV_ equals JC and *DSC*_EV_ equals DSC). The limitations of this study include: (a) due to the irregular nature of dose coverage, the dose distance calculated based on the equivalent sphere is only a rough estimate and may not fully reflect the actual clinical situation; (b) the reliability of the proposed method needs to be further validated across a broader range of disease types and with larger sample sizes; (c) the recommended values (*n* = 1, *d_r_* = 1 cm) should be regarded as practical settings for the present NPC/VMAT dataset, and their applicability to other disease sites or more anisotropic dose‐gradient scenarios remains to be further validated.

## 5 CONCLUSION

This study validated a strong negative linear correlation between the peripheral dose and the distance from the target volume. Although the regression coefficient varied with PTV size, the observed inter‐case variability was low, and the linear relationship was consistently robust across all individual cases. A reference penalty distance of 1 cm, combined with a distance‐penalty parameter n of 1, is proposed as a clinically practical and reasonable configuration for the *CC*_EV_. The *CC*_EV_ framework offers a comprehensive solution by fully accounting for the spatial location of non‐overlapped volumes and incorporating key radiotherapeutic requirements. Its design is both versatile and generalizable, as it can be simplified to other standard indices, making it a robust tool for future applications in target volume comparison.

## AUTHOR CONTRIBUTIONS

**Xue Ou**: Data curation; project administration; writing—original draft. **Xianfei Qin**: Data curation; methodology; project administration. **Huaye Wei**: Formal analysis; investigation. **Di Huang**: Investigation; project administration. **Lianrong Zheng**: Data curation; formal analysis. **Qiulu Zhong**: Data curation; and investigation. **Qinghua Du**: Conceptualization; formal analysis; writing—review; and editing. **Kai Hu**: Conceptualization; formal analysis; writing—review; and editing.

## CONFLICT OF INTEREST STATEMENT

The authors declare no conflicts of interest.

## DATA AVAILABLITY STATEMENT

Data will be made available on request.

## ETHICAL APPROVAL

This retrospective study was reviewed and approved by the Institutional Review Boards of The Second Affiliated Hospital of Guangxi Medical University.

## CONSENT

The requirement for individual informed consent was waived.
